# *Pelodera*: cosmopolitan phoretic saprotrophs and neglected models for origins of nematode parasitism

**DOI:** 10.1186/s13071-025-07041-1

**Published:** 2025-11-21

**Authors:** Paul M. Airs

**Affiliations:** 1 Queen’s University Belfast, Belfast, UK; 2 Murray Edwards College, University of Cambridge, Cambridge, UK

**Keywords:** Phoresy, Dauer, Necromeny, Soil health, Scarabaeidae, Beetle, Decomposition, Decay

## Abstract

*Pelodera* (A. Schneider, 1866) is a Clade V nematode genus of the Rhabditidae family and close relative of *Caenorhabditis elegans*. The genus comprises 26 species poised between free-living, commensal, and parasitic lifestyles, as well as species with underappreciated roles in soil nutrient cycling. *Pelodera strongyloides*, the type species, is a cause of follicular larva migrans among humans and other mammals but can be confused with hookworm folliculitis. With minimal genetic resources available, the capacity to identify and diagnose infectious species and strains of *Pelodera* are limited. Thus, the ecology of *Pelodera* species as both parasites and commensalists is likely underreported and possibly mischaracterised. Similarly, decomposition biome studies report *Pelodera* and other Rhabditidae as dominant taxa during decay but require greater genetic resources to classify species. To renew appreciation for these neglected model organisms, this review collates available literature to detail *Pelodera* associations across mammalian and invertebrate hosts and discusses traits that drive host association such as dauer formation, waving (nictation), phoresy, and tissue invasion. The potential for *Pelodera* as a satellite model to *C. elegans* is also discussed since *Pelodera* are readily culturable while having inducible parasitic forms. Research into *Pelodera* will not only improve understanding of their ecology and contribution to decomposition but may prove invaluable in identification of behaviours underpinning parasitism and parasite origins. Utilising *Pelodera* facultative parasites may be essential to triangulate and resolve differences between *C. elegans* and obligate nematode parasites with similar niches such as hookworms, lungworms, *Strongyloides*, and gastrointestinal nematodes of livestock.

**Graphical Abstract:**

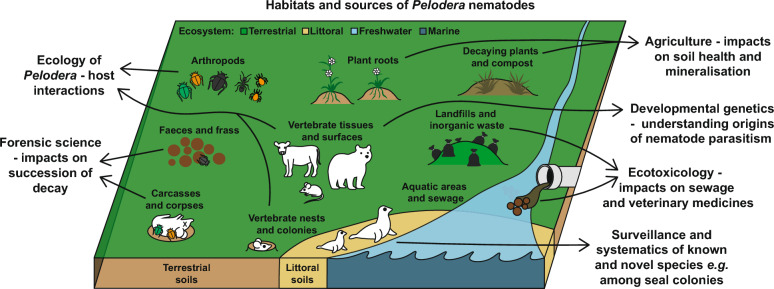

## Background

**Fig. 1:**
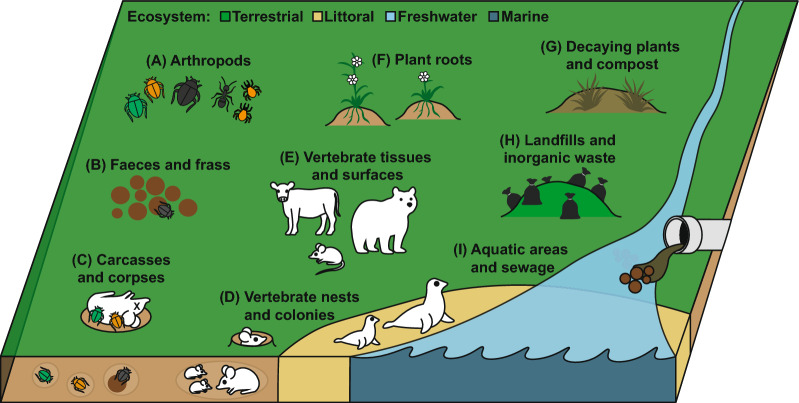
The interconnected habitats and hosts of *Pelodera* species. *Pelodera* species have been isolated from a number of habitats and associated hosts cross referenced in Table 1. These include, (A) tissues, surfaces, and frass of living and dead arthropods, (B) faeces and frass of vertebrates, (C) vertebrate carcasses and human corpses, (D) vertebrate burrows, nests, moulting grounds, and colonies, (E) vertebrate infections and tolerated phoresy, particularly of skin, (F) roots and rhizospheres of plants, (G) dead organic matter of plants and other non-animal sources, (H) landfills or sites of human generated waste, (I) aquatic areas and sewage

*Pelodera* (A. Schneider, 1866) is a cosmopolitan genus of rhabditid nematode with species occupying a wide range of habitats from fresh water, soil, dung, and mammalian hosts (Fig. 1, Table 1). Host associations are a common feature, with many species displaying different degrees of ‘phoresy’, where invertebrate and vertebrate hosts are used for transport to nutrient rich habitats [1, 2]. Such associations also enable a degree of parasitism witnessed in some species, although the relationships *Pelodera* species have with their hosts still require much more attention to be properly understood. For the parasitic species, these are thought to lie somewhere between phoretic, facultative parasitism, and ‘fortuitous parasitism’ (as described by Anderson [3]). Despite potentially long evolutionary distances between closely related species of nematodes [4], all described *Pelodera* appear to be bacterivore saprophytes for at least a portion of their life cycles, for instance *Pelodera punctata* (syn: *P. chitwoodi*), which is found in aquatic sewage sludge [5, 6], preferentially migrates toward and consumes *Vibrio* bacteria [7].

**Table 1:** Species and habitats of the genus *Pelodera*

Clade^a^	Species	Description^b^	Nemys TaxID^c^	Sources as described in descriptions	System Figure 1^d^	Habitat Figure 1^d^
S	*P. arnbomi*	Boström (1996)	1537654	Organic matter from an elephant seal moulting ground	Littoral	D, E*
S	*P. comandorica*	Belogurov (1977)	1537655	Marine littoral zone of Commander Islands	Littoral	D*
S	*P. cutanea*	Sudhaus (1987)	1537847	Skin of rodents including wood mice (*Apodemus*)	Terrestrial	B, D, E
S	*P. litoralis*	Skwarra (1921)	1294689	Carrion of crow (*Corvus*)	Terrestrial	C
S	*P. merionis*	Sudhaus (1991)	1537848	Nests and skin of gerbils (*Meriones tamariscinus*)	Terrestrial	B, D, E
S	*P. nidicolis*	Sudhaus (1986)	1537849	Nest of a field vole (*Microtus agrestis*)	Terrestrial	B, D, E*
S	*P. orbitalis*	Sudhaus (1986)	1537850	Field vole (*Microtus agrestis*) and other rodents (Muridae, Arvicolidae), dauer juveniles parasitic in the conjunctival sac	Terrestrial	B, D, E
S	*P. punctata*	Cobb (1914)	1294690	Roots of aquatic plants in Potomac River; also in decaying deposits on the shore of fresh water bodies, and in sewage sludge	Freshwater	F, G, I
S	*P. strongyloides strongyloides*	Schneider (1860)	1538808	Decaying organic matter, moist soil. Not-parasitic, dauer juveniles do not wave	Terrestrial	B, G
S	*P. strongyloides dermatitica*	Sudhaus (1988)	1538806	Dauer larvae invade the skin and hair follicles of various mammals including humans causing follicular larva migrans. Common in soils	Terrestrial, Littoral?	A, B*, D, E
S	*P. termitis*	Carta (2010)	1537656	Head capsules of termites (*Anacanthotermes turkestanicus*)	Terrestrial	A
C	*P. adeeli*	Mahboob (2023)		Encysted on dung beetle (*Onthophagus ramoss*)	Terrestrial	A
C	*P. aligarhensis*	Tahseen (2014)	1586699	Terrestrial landfill	Terrestrial	H
C	*P. coarctata*	Leuckart (1891)	1537840	Encysted on European dung beetle (*Aphodius fimetarius*), cow pats	Terrestrial	A
C	*P. cylindrica*	Cobb (1898)	1537841	Faeces, compost, and decaying fruit. Associated with roots and rhizosphere of tomato (*Solanum lycopersicum*)	Terrestrial	F, G
C	*P. cystilarva*	Völk (1950)	1537842	Encysts on various arthropods (particularly Macrocheles mites and ants), as well as rotting plants, rotting wood, and compost	Terrestrial	A, G
C	*P. indica*	Mahboob (2023)		Encysts on dung beetles (*Catharsius molossus*)	Terrestrial	A
C	*P. isociensis*	Maupas (1916)	1537843	Encysts on Earth borer beetles (Geotrupes), faeces, and mould	Terrestrial	A, B, G
C	*P. kolbi*	Sachs (1950)	1537844	Cow pats	Terrestrial	B
C	*P. operosa*	Andrássy (1962)^†^	584046	Associated with roots of barley	Terrestrial	F
C	*P. par*	Andrássy (1962)	584047	Horse manure	Terrestrial	B
C	*P. paratretzeli*	Mahboob (2023)		Encysts on dung beetles (*Digitonthophagus bonasus*)	Terrestrial	A
C	*P. serrata*	Körner (1952)	1294691	Scarab beetle frass, dauer juveniles form cysts that are attached to insects by secretion from the amphids	Terrestrial	A
C	*P. tretzeli*	Sachs (1950)	1537845	Cow pats	Terrestrial	B
C	*P. voelki*	Sachs (1950)	1537846	Cow pats	Terrestrial	B
T	*P. parateres*	Cobb (1924)	1537852	Slimy celery; mainly in compost	Terrestrial	G
T	*P. pseudoteres*	Schulte (1989)	1537853	Encysts on carrion beetle (*Nicrophorus humator*); owl pellets, slime flux on trees, dung; dauer juveniles are waving	Terrestrial	A, B, G
T	*P. scrofulata*	Tahseen (2014)	1586700	Fresh water, sewage drains	Freshwater	I
T	*P. teres*	Schneider (1866)	1294692	Wide range of saprobic habitats. Faeces, human corpses, rotting vegetables	Terrestrial	B, C, G
	*P. parasitica*	von Linstow (1907)^†^	1537854	In buccal pouch of African bush pig (*Potamochoerus larvatus*) alongside wood	Terrestrial	E*, G*

(a) Clades indicate S = strongyloides, C = coarctata, and T = teres

(b) As listed by Sudhaus and Mahboob [2, 13, 16]. ^†^Species provisionally accepted by Sudhaus [2, 13]

(c) From the world database of nematodes: https://www.nemys.ugent.be/

(d) Ecosystem and habitat as detailed in Fig. 1

* indicate a presumed ecological niche

Morphologically, *Pelodera* are ‘Pleiorhabditis’ (species with a posterior vulva), are gonochoristic (sexual dimorphism with male-female cross-fertilization required for reproduction), and exhibit a ‘peloderan’ type male retracted tail [4, 8]. All of these traits have a high level of convergent evolution among Rhabditida and have passed through multiple rounds of gain and loss in some species. The gain and loss of different features are to be expected considering the possibility that nematodes have ancient points of divergence or have an advanced rate of genetic mutation or recombination [4].

*Pelodera* are members of the ‘Rhabditidae’. While this group is paraphyletic, the genus *Pelodera* is both morphologically and phylogenetically monophyletic [4, 9]. The genus has, on numerous occasions, been subject to update by Dougherty (1953), Andrássy (1976), and several times by Sudhaus (2011 and 2023) as part of wider revisions of the Rhabditidae [2, 10–13]. As a result, *Pelodera* has also been subject to significant revision, but many species have remained over time despite having somewhat disparate ecologies [including the type species *Pelodera strongyloides* (Schneider, 1860)]. 

In Sudhaus’ 2011 and 2023 works, *Pelodera* is defined as a genus with three monophyletic clades including the sometimes parasitic ‘strongyloides’ (11 species), the dung-beetle associated ‘coarctata’ (11 species), and the compost associated ‘teres’ groups (4 species) [2, 13]. Hodda’s 2022 classification of Nematoda places *Pelodera* in the subfamily Peloderidae (Andrassy, 1976), tribe Peloderini (Andrassy, 1976), and subtribe Peloderinii (Andrassy, 1976), wherein 16 species of *Pelodera* exist, with 9 species among the sister genus *Coarctadera* and one species in the sister genus *Rhomborhabditis* [21]. However, the World Database of Nematodes recognises 26 species, including two subspecies of *Pelodera strongyloides* (*dermatitica* and *strongyloides*) and a provisionally accepted *Pelodera parasitica* [14]. New species are still being discovered, and much is still unknown about the diversity and distribution of the genus [15, 16]. For the purposes of this review, the 26 species described by Sudhaus (2011 & 2023) and the World Database of Nematodes are included alongside three newly described species of *Coarctata* (Table 1) [2, 13, 14, 16].

That so many closely related species fill so many different niches makes them interesting from a comparative analysis standpoint. This is particularly true when studying developmental plasticity, cues for taxis, cues for development (especially phoresy and dauer), and cues that initiate host associations.

## *Pelodera* ecology and evolution—phoresy, necromeny, facultative-parasitism, or parasites in transition?

### Soil nutrient cycling and ecosystem services of *Pelodera* as detritivores

*Pelodera* species descriptions come from a wide range of habitats and appear to colonise both living and dead animals and plants (Fig. 1, Table 1). Given their presence and prevalence among soils and substrates worldwide, *Pelodera* may be an important part of establishing soil communities. Pelodera are most commonly associated with nutrient-rich organic materials such as faeces, carrion, and compost so they may be a key link in the succession of decay (see “Phoresy and the ‘dauer hypothesis’—an adaptation enabling colonisation of new hosts” section). Among soils, *Pelodera* have been shown to improve nitrogen mineralisation through bacterial grazing and release of NH4+–N, which improves early plant root growth compared to bacteria or bacteria with fungal feeding nematodes [17]. In this study, Ingham et al. also identified that *Pelodera* increase plant shoot phosphorus when active in the soil. More generally, nematode communities are known to be essential to soil function through mineralisation of nitrogen and phosphorus, balancing bacterial and fungal activity, and improving plant growth, critically impacting crop yield and pasture health [17–21]. *Pelodera* species may therefore be underappreciated as indicators of good soil health since they are largely understudied and there is limited genetic information available. Modern analyses rely on bioinformatic taxonomic information to classify the presence and abundance of different species in large microbiome datasets, but many species lack genetic barcodes (see Table 2). From available species data, however, it is clear that *Pelodera* are significant members of soils, especially in cultivated areas [22, 23]. A recent and novel topological framework to analyse taxa driving soil health (gFlora) identified *Pelodera cylindrica* to be one of the five most important soil species for potential nitrogen mineralization [23]. This work also identified that *Pelodera cylindrica*, *P. strongyloides dermatitica*, and *P. teres* make up three of the seven most important nematode taxa that enhance crop yield. More study is still needed however to determine the ecological services provided by *Pelodera* in nutrient cycling and soil establishment.

**Table 2:** Available sequence information for molecular biobanking and phylogenetic classification of *Pelodera* species

Clade^a^	Species	NCBI TaxID^b^	Available sequence data^c^
S	*P. punctata*	96649	4 rRNA and RNAP sequences
S	*P. strongyloides strongyloides*	35557	Genome (GCA_036852015.1), 12 rRNA and 1 COI sequence
S	*P. strongyloides dermatitica*	35558	3 rRNA and RNAP sequences
C	*P. cylindrica*	479152	2 rRNA sequences
T	*P. pseudoteres*	473159	6 rRNA and RNAP sequences
T	*P. teres*	96650	6 rRNA and RNAP sequences
	Unspecified* Pelodera*	2620560	12 rRNA sequences

(a) Clades indicate S = strongyloides, C = coarctata, and T = teres

(b) From https://www.ncbi.nlm.nih.gov/Taxonomy/Browser/

(c) Sequences including ribosomal RNA (rRNA), RNA polymerase (RNAP), and cytochrome oxidase subunit 1 (COI). As of July 24 2025

Beyond being saprotrophs and bacterivores, there may be secondary impacts of *Pelodera* on soil establishment through host-attachment (see “Waving behaviour—a form of nictation that facilitates host attachment; Ectophoresy and invertebrate associations; Endophoresy and necromeny—entering the host; Parasitism” sections). The impacts of *Pelodera* on other members of the nutrient-cycling community such as carrion beetles and dung beetles, to which many *Pelodera* can attach, are relatively unknown. There is a growing appreciation of ecosystem services carried out by detritivores such as dung beetles, which improve soil health and bury gastrointestinal nematode parasites among ruminant pastures [24, 25]. However, the impact of nematodes such as *Pelodera* on Scarabaeidae requires further study, especially in the context of farming, where nematocidal and insecticidal compounds are routinely applied as part of production (see “Technology transfer and functional validation of parasitic behaviours” section).

### Phoresy and the ‘dauer hypothesis’—an adaptation enabling colonisation of new hosts

**Fig. 2:**
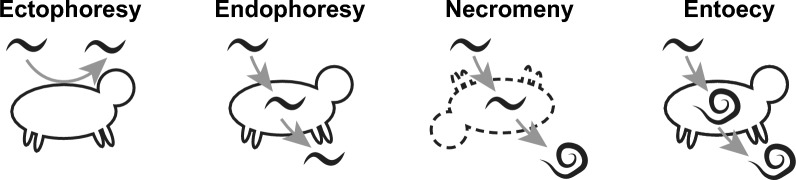
Forms of phoresy among nematodes. A number of terms exist to describe the use of hosts for phoresy among nematodes. These include: ectophoresy, where L3 temporarily attach to surfaces of living hosts, endophoresy, where L3 temporarily enter and exit from living host cavities or tissues, necromeny, where L3 enter hosts and remain dormant until death of the host, then complete their life feeding on bacteria or necrotic host tissue, and entoecy, where L3 complete life cycle within living hosts without causing noticeable harm or depriving nutrition. It may be that several different behaviours are possible for any given species and more detailed studies are required to better understand the ecology and functions of each

The concept of phoresy, where organisms ‘hitchhike’ on hosts to get around, is not unique to nematodes and has recently been subject to review [1, 26]. Among nematodes, phoresy is a common behaviour, with 40,000–500,000 species of insect-associated nematodes thought to exist, many of which are phoretic [27]. The act of phoresy has a number of advantages for nematodes, mainly to reach new sources of nutrients and to be protected against climatic stressors [1, 28]. Host associations also enable translocation many orders of magnitude greater than physically possible alone and can therefore facilitate colonisation of new areas or increase the likelihood of contact with other populations of the same species to prevent genetic bottlenecks [29]. With so many species developing this trait of dispersal, it is perhaps unsurprising that phoresy has diverged with the act of host association playing numerous ecological roles (Fig. 2). The phoront (organism using phoresy as a behaviour) may benefit from translocation not only as a means of dispersal but also as a delivery system to fertile grounds. For instance, the various *Pelodera* species associated with saprobiotic beetles benefit from phoresy since beetles will bring them directly to feed sources such as bacteria in decomposing dung (see Table 1). Hosts may also provide a source of nutrients, such as rodent-associated species, which feed on bacteria growing in the faeces and frass of their hosts. The host itself can also be a source of food if phoretic nematodes are present on or in the host at the time of death. Detailed below, this form of phoresy is dubbed necromeny, and species such as *P. strongyloides* have been found to feed on dead beetle and rodent hosts [30, 31].

Since hosts can become intertwined with sources of nutrition, the jump from ‘free-living’ to vertebrate parasitism is also thought to begin through phoresy [1, 26, 27, 32]. As a behaviour, phoresy in nematodes is linked to dauer formation and the act of waving (nictation), such as in *Caenorhabditis elegans* [33]. The developmental plasticity of dauer and nictation behaviours may open the door to parasitism among many nematodes, particularly Clade V species. Sudhaus describes this process for the Secernentea, stating that “a transformed dauer larva initiated special associations like endophoresy, entoecy, necromeny, larval parasitism, accidental, facultative and obligate parasitism” [32].

In *C. elegans*, dauer formation enables tolerance and survival in unpredictable pre-living environments when conditions are too harsh for normal development, such as extreme temperature, nutrient deficits, or high population densities [34–36]. In this case, dauer is a conservative approach that effectively arrests development [36, 37]. However, dauer states also underline host-seeking and phoretic associations that are a pre-adaptation facilitating parasitism in many Clade V species, including *C. elegans* [34, 38]. The ‘dauer hypothesis’ states that the unpredictability of free-living habitats leads to a high level of adaptability, resulting in dauer states, which over time become ‘infective’ life stages [34, 38]. Much of the dauer pathway is also conserved between parasitic and free-living species, but tracing individual cues that induce parasitic behaviours have been difficult to underline [34].

*Pelodera* species may provide insights into the early stages of divergence between free-living and parasitic lifestyles with many specific and general hosts employed. For instance, *Pelodera orbitalis* has a high level of developmental plasticity, with inducible parasitic, free-living, and dauer L3 stages [39]. This ‘triphenic’ capacity deserves attention as the formation of dauer vs. infectious L3 stages is interesting as a point of parasitic origin [32, 34, 40]. The inducible nature of infective and dauer states in *P. orbitalis* may therefore give a better understanding of the function of dauer in facultative parasites [39]. However, there is still much to discover about dauer formation, particularly concerning conserved and divergent elements between free-living and parasitic species [34]. In *C. elegans*, developmental switching to the dauer state is mediated by the dafachronic acid steroid hormone acting through its nuclear receptor DAF-12 [35, 41]. This receptor and many of the upstream signalling pathway components are intact across a broad range of parasitic nematodes [34, 42]. However, distinctions exist such as for the TGF-β ligand DAF-7. DAF-7 functions upstream, regulating dafachronic acid production, and thus represents a major entry point for dauer regulation in *C. elegans*; daf-7 expression is inversely associated with the infective larval stages of hookworms [43, 44] and Trichostrongylid nematodes [45]. This is also seen in *P. strongyloides* where a recent transcriptomic analysis did not identify *daf-7* (B0412.2) or indeed any of the daf genes to be upregulated in starved dauer state larvae but did identify other dauer entry genes such as *prmt-1* (Y113G7B.17) [46] to be upregulated while *tatn-1* (CE10298) was downregulated. In *C. elegans,* DHS-28 is an upstream regulator required for dauer pheromone biosynthesis [47], but in *P. strongyloides dhs-28* (CE04770) was downregulated in dauer larvae [48] (personal wormbase mine of supplemental data files). Also, starved *P. strongyloides* had downregulated *lin-12* (CE00274), a dauer exit gene in *C. elegans* [49]. Taken together, these data highlight divergence in the dauer pathway between *Pelodera* and *C. elegans* despite their relative evolutionary proximity compared to more distant parasites. The specific genetic switches driving dauer formation vs. infectious state L3 have yet to be identified but may shed light on infectious L3 development in obligate parasites (see Box 1 points 8–10). There is a growing body of genetic and transcriptomic evidence of conserved roles between *C. elegans* and parasitic species, but the complexity of parasitic nematode life cycles and a lack of developed in vitro systems limits validation or functional data [34]. Again, *Pelodera*, being at a crossroads between free-living and parasitic, may provide an avenue to chase specific questions relating to pre-existing traits priming parasitic lifestyles, such as entry to dauer and infective larval states, and are worthy of cross-comparative study.

### Waving behaviour—a form of nictation that facilitates host attachment

Nictation, or the act of waving, is a behaviour conserved in free-living nematodes and phoretic nematodes [33, 50], which additionally facilitates attachment to hosts for infectious species [40, 51]. *P. orbitalis*, which live in close association with rodents and infect the ocular sacs, exhibit waving among infectious L3 to attach to their hosts [39]. *Pelodera cutanea*, which infects the skin of wood mice, is similar and has ‘waving’ exsheathed infectious L3 that are more active than the “relatively clumsy”, “coiling up when disturbed”, and sheathed dauer larvae [52]. In the case of *Pelodera pseudoteres*, L3 larvae were witnessed waving when in the presence of dried meat [53]. This behaviour was extreme, and Schulte noted a ‘flame’ where clusters of individuals formed one large structure which waved in unison. This behaviour is similar to ‘swarming’ seen in *C. elegans *[41]. However, this behaviour has not been reported in other descriptions, and further study is needed to better understand the function of this behaviour since *P. pseudoteres* is usually not host-associated.

Dispersal activity patterns can also vary periodically with periods of intermittent dormancy. Species that are dependent on hosts for nutrients must also rely on dispersal behaviours that maximise periods of activity while minimising risk of stress. For instance, hookworm larvae lay in wait, conserving energy until any one of a number of host cues triggers active taxis toward a host [54]. This tactic is not conserved to nematode parasites and can be seen in ticks, which display ‘questing’ behaviour, with individuals climbing grass or substrates and waiting for hosts, and then actively grabbing and moving onto anything that passes by [55]. In *Pelodera*, waving is associated as a distinct state in infectious L3 and not seen in dauer larvae (except for some species like *P. pseudoteres*). While the developmental cues inducing infectious L3 states have yet to be resolved, such as for *Strongyloides* species [56], there is a wide range of known cues that induce waving among a number of species. These include abiotic factors such as heat and CO_2_ as well as biotic factors such as microbes or volatile compounds (reviewed by Gang and Hallem [51]). *Pelodera* may prove useful in determining the biology of waving and stimulation of this behaviour in Clade V species [57]. For instance, thermal gradients activate host seeking of *P. orbitalis* parasites of rodents [39]. Similarly galvanotaxis (migration under an electric field) has also been shown for *P. strongyloides* larvae which migrated toward an anode, which may mimic the electric field of mammalian hosts [58].

### Ectophoresy and invertebrate associations

Ectophoresy is the most basal physical association between a nematode and a host where nematodes adhere to the ectodermal surfaces. *Pelodera* species display ectophoresy with insects across five insect families but are particularly associated with the legs of dung and carrion beetles (Scarabaeidae) [16, 59]. At least 9/26 of described *Pelodera* species are insect associated (Table 1). This includes the parasitic *P. strongyloides*, which has been found attached to dead stag beetles (*Lucanus ibericus*) [31]. Ectophoresis is also common among the coarctata clade, with dauer juveniles of *Pelodera adeeli*, *P. coarctata*, *P. cystilarva*, *P. indica*, *P. isociensis*, *P. paratretzeli,* and *P. serrata* found encysting on insects and other arthropods (Table 1).

What is unusual is the predominance of *Pelodera* among beetles, with hundreds of *Pelodera* larvae isolated from the legs of single dung beetles, far more than for other phoretic nematodes [31, 60, 61]. This may be due to the prevalence and abundance of *Pelodera* among soils overall [23]. In addition to beetles, *Pelodera* have also been found in association with termites [62] and Macrochelid mites [63]. Both invertebrate and mammalian associations likely function as dispersal mechanisms, transporting *Pelodera* colonies to new nutrient rich habitats [3, 40, 50] (Fig. 2). Host-attachment behaviour may even be beneficial to dung beetle and other invertebrate hosts by aiding decomposition of faeces and consumption of potentially infectious bacteria, but much is unknown relating to host-*Pelodera* relationships.

Ectophoretic host associations are not random and are facilitated by production of specific sticky substances that have yet to be defined [64]. Such behaviours are signalled by specific cues [27]. For instance, *Caenorhabditis japonica* are attracted to hexane-extracted kairomones (allochemicals that induce positive responses, e.g. chemotaxis) of burrower bugs *Parastrachia japonensis* [65]. Similarly, various fig nematodes specifically detect and are attracted to volatiles and cuticular hydrocarbons of pollinating fig wasp species and are not attracted to other non-pollinating fig wasps [66]. *Pristionchus* species also use volatiles and cuticular hydrocarbons emitted by their hosts as chemoattractants [67]. In the case of *Pristionchus pacificus*, a satellite model for *C. elegans*, the host scarab beetle (*Exomala orientalis*) sex pheromone (Z)-7-tetradecen-2-one induces phoretic behaviour and host attachment [68].

Ectophoretic associations are also critical for parasitic and pathogenic nematodes, particularly plant-parasitic nematodes such as the pinewood nematode *Bursaphelenchus xylophilus*, which detects and attaches to eclosing Monochamus pine sawyer beetle vectors in order to spread to new tree hosts [69]. Beyond chemical associations, some species have found spatial niches on their phoretic hosts. For instance *Caenorhabditis drosophilae*, which attaches to an inflatable sac (the ptilinium) on the head of *Drosophila* hosts [70].

### Endophoresy and necromeny—entering the host

From ectophoresy, it is a small jump to invade host tissues and achieve endophoresy. Endophoresy is defined as commensal colonisation of the host tissues without causing disease. Examples include some *Caenorhabditis* species with some found in strange places such as *Caenorhabditis remanei* wedged in dorsal plates woodlice [71]. *Pelodera cystilarva* is among a small group of ‘myrmecophilous’ nematodes which can infest the postpharyngeal glands (or other structures) of ants and may be a specialised component of ant biomes [64]. Specialisation to enter the pharynx of ants is specific to *P. cystilarva*, since other species such as *P. strongyloides* have been shown incapable of infesting ants under experimental conditions [72]. *Pelodera termitis* goes one further and has been found inside the head capsules of termites in Turkestan termites (*Anacanthotermes turkestanicus*) [62]. In both of these cases, entering the host can occur potentially through ingestion, so endophoresy could have emerged as a result of these species being present among the same feeding grounds as their hosts, i.e. rotting wood [64].

The difference between endophoresy and necromeny is the persistence and dormancy of embedded larvae until the death of the host. Dormancy is a necessity that can enable organisms to withstand periods of stress [36], so there may be differential cues that lead to necromeny vs. endophoresy. Endophoresy implies nematodes only remain within their hosts for a short time span, to exit once arriving at a new habitat. Necromeny however implies that the destination is less important, since the host doubles as a feeding ground following its death [32]. *Pelodera* is thought to exhibit necromeny of rodent hosts (see Table 1). For instance, some species exit their rodent hosts following a drop in host body temperature [30]. Sudden drops in body temperature may be a signal that the host has died and therefore this adaptation may be a programmed response for necromenic behaviour. In this scenario, consumption of the host is also not parasitic if the phoretic nematodes do not lead to the death of the host, and *Pelodera* are thought to be well tolerated by rodent hosts [32, 73, 74]. Typically, necromeny exists among bacterivores who do not consume host tissue directly but consume the microbiome of decaying hosts, such as for *Pristionchus* species [61, 75]. For *P. pacificus*, necromeny occurs as encysted larvae leave the host a week after death to feed on bacterial decomposers. This is mildly convoluted for entomopathogenic nematodes like *Heterorhabtidis* and *Steinernema*, who carry bacteria that cause disease and kill their insect hosts [76]. While not strictly parasitic, this tag team has found a niche for itself at the expense of their hosts through the introduction of pathogenic bacteria. There is much to be learned about the role of *Pelodera* as part of the mammalian and insect ‘necrobiome’. For instance, *Pelodera* have been found to be overwhelmingly abundant on beaver carcasses (*Castor canadensis*) [77]. In this study, Diplogasteridae (relatives of *Pristionchus*) were more abundant earlier, on day 15, while *Pelodera* came later and were the dominant taxa at day 40. However, numerous Rhabditida present in early stages of pig (*Sus scrofa domesticus*) decay are replaced during bloat, only to return as the most abundant eukaryote during advanced decay [78]. Rhabditida also dominate summer grave soils of mammal carcasses but not control soils [79]. The close relative and Rhabditid *Oscheius tipulae* has also been found to dominate grave soil, head, abdominal, and skin tissues of decaying mice [80]. Unfortunately, most studies looking into the succession of decay are unable to distinguish *Pelodera* from other Rhabditid species owing to a lack of molecular reference data, and so it is impossible to appreciate the ecological role of *Pelodera*, but it may be that some species specialise in colonising sick or dying hosts and initiate rapid decomposition post-death. Alternatively, *Pelodera* abundant in the soil could rapidly invade carcasses following death.

There remains limited knowledge linking necromeny of *Pelodera* species to decomposition of cadavers, i.e. we do not know whether *Pelodera* species present during decomposition originate in the soil or were previously embedded in host tissues. *Pelodera* are found among wildlife that are in very poor condition [81–85]. Therefore, it may be that *Pelodera* infections are linked to immunosuppression. However, *Pelodera* infections result from exposure to contaminated mud or soil [84, 86]. Therefore, the environmental pollution or exposure to other pathogens may lead to *Pelodera* infections rather than *Pelodera* taking advantage of ‘near death’ individuals in which to invade and undergo necromeny. Determining the order of events will be key to improved understanding of *Pelodera* ecology and the potential benefits of *Pelodera* infections for rapid decomposition and limitations of disease spread.

### Parasitism

As discussed above in necromeny, the journey to direct parasitism or pathology may be a simple case of continued survival while already encysted within host tissue. A number of *Pelodera* species are confirmed or potential parasites, including ‘strongyloides’ clade *P. strongyloides, P. orbitalis, P. cutanea, P. merionis, P. nidicolis* and the ‘teres’ clade *P. pseudoteres* (Table 1) [2, 13]. As discussed above, whether these species are ‘endophoretic’ or ‘parasitic’ will depend on the degree of pathology caused. It may be that these species are endophoretic or necromenic rather than parasitic as they do not appear to cause harm to their rodent hosts, or at least are well tolerated [30, 32]. However, all these species display waving nictation behaviours and probably have ‘infectious’ L3 states. Without further study, the degree of parasitism and facility to cause zoonoses remains unknown for many of the ‘strongyloides’ clade of *Pelodera* and *P. pseudoteres*.

Parasitism among predominantly soil-dwelling nematodes may have the advantage of persisting over time or long distances in areas where nutrients are sparse or strongly seasonal. For example, species distributions are widespread and include remote areas such as *Pelodera arnbomi* on the subantarctic island of South Georgia and *Pelodera comandorica* on the Commander Islands in the Bering Strait [87, 88]. The presence of *Pelodera* species in such remote locales may be due to the parasitism of seals, since both *P. arnbomi* and *P. strongylodies* have been identified among seal colonies and *P. strongyloides* is a known parasite of mammals [87, 89]. An association with seals would facilitate the movement of *Pelodera* to areas of seal activity and essentially improve the decomposition of frass and faeces, potentially benefiting the seal colony as well as establishing soils in littoral zones. However, both *P. comandorica* and *P. arnbomi* are poorly studied, so it remains unknown if these species use seals as hosts, cause any harm to seals, or if marine mammals as a form of nematode zoochory.

To invade hosts, *Pelodera* species require passage into tissues. Dermal layers are not a typical endpoint for endoparasites, since the skin is the first layer of defence and leaves parasites more or less exposed. However, examples do exist. Cuterebra botflies are a classic case, where larvae actively parasitise their hosts, cause harm, take nutrients, and undergo development before exiting [90]. For nematodes, a wide range of species, including *Pelodera, Strongyloides*, hookworms, filarial nematodes, *Spirulina*, and * Gnathostoma,* can cause diseases such as cutaneous larva migrans and folliculitis [91–95]. Typically, skin invading nematodes (including vector borne species) traverse the dermis and migrate to more suitable niches such as the lymph nodes and peritoneal cavity (e.g. *Wucheraria bancrofti* and *Mansonella* spp.), intestinal tract (e.g. intestinal hookworms and *Strongyloides stercoralis*), and subcutaneous tissues (e.g. *Loa loa* and *Onchocerca volvulus*) [3, 96, 97]. For *Pelodera* species, the cause of parasitism is mainly through follicular larva migrans [91]. In such cases, the L3 stage of *P. strongyloides dermatitica* invades follicles and undergo hypobiosis, causing rashes and papules in their mammalian hosts [92]. Follicles are seen as a major route for human hookworms to invade deeper tissues [98], so the emergence of *Pelodera* disease could occur with sufficient stress and time.

Generally, however, L3 larvae of *Caenorhabditis* and *Pelodera* species are non-feeding and do not grow during dermal invasion. This is not always true, since osmotic feeding of conjunctival sac secretions occurs during *P. orbitalis* infections [39]. The principal function of *Pelodera* invading host tissues appears to be necromenic, especially since larvae exist in dead hosts rather than feeding directly on host tissue [30]. For instance, larvae extracted from medical cases have developed following culture on meat, but these larvae did not develop in the host [99]. Other species, such as *P. pseudoteres*, can develop on meat, but it is unknown whether the consumption is of mammalian tissue or on bacteria that grow on the tissue [53].

For both *Caenorhabditis* and *Pelodera*, the presence of free-living and parasitic species coexisting within similar habitats provides a rare opportunity to identify differences facilitating parasitism. This comes with the possibility of tracing the origins of parasitism in species which appear to only superficially impact their hosts. For *Caenorhabditis bovis* (found in the inflamed ears of Zebu cattle), these facts prompted genomic investigation [100]. Since many *Pelodera* species are also exclusively free-living [2], cross-comparisons between *Pelodera* might prove useful in identifying the genetic basis of parasitic lifestyles.

## The many utilities of *Pelodera* research

A brief history and future for non-model nematode research Parasitic species were once the highlight of nematological study because of their diverse and complex biology [101], a fact that has become a hindrance due to the complexity of their behaviours and host requirements, which together limit capacity for modern study. Free-living *C. elegans*, *Caenorhabditis briggsae*, and * P. pacificus*, among other free-living species, have since become models for study, demoting parasites such as *Ascaris suum* that were of greater interest in the nineteenth century [102]. Being readily culturable, having a large scientific community, and being amenable to mutagenesis, *C. elegans* has become not only a model for the study of free-living traits but also a stand-in for parasitic species [103, 104]. ‘Culturability’ is key for the study of nematodes and has been a major roadblock to studying diverse parasitic species. As a result, tools developed in *C. elegans* can also dictate the technology being applied to parasitic species, with techniques always being trialled and adapted to parasitic species [105]. Nevertheless, the rich history of nematology has left us with a number of excellent and comprehensive descriptions that continue to be updated, including introductions to nematology [101, 106], compendiums of nematode anatomy [107, 108], and encyclopaedias of parasitic nematodes of vertebrates [3, 109] and plants [110]. These volumes cover aspects of *Pelodera* and the wide diaspora of relatives. While invaluable to their respective fields, significant knowledge gaps exist between ‘wild’ species and their model counterparts. This gap has been growing steadily over time with the scope for research narrowing to a smaller set of specific model species. This narrow scope allows for deeper understanding of form and function at the cell and tissue levels [111]. The downside of the modern era is the dearth of interest in maintaining and updating knowledge relating to basic biology among the non-model species that significantly diverge from models [4]. Even the most well-studied parasitic nematodes lag behind *C. elegans* studies in breadth and depth.

For *Pelodera*, early laboratory studies have not been updated in the past 30–50 years, even though there is good evidence of *Pelodera* being ripe for laboratory studies [57]. Furthermore, little to no genetic resources are available (Table 2), although genome and transcriptome sequences have been made, also coming from the Sanger Institute for *P. strongyloides* [48, 112, 113]. The situation for basic research of parasitic nematodes is generally dire across the board, leading to calls for updated evaluations and basic studies, yet these are scarcely funded [114]. The characteristics of *Pelodera* might aid this effort, requiring minimal resources because of the simplicity of culture [30, 115, 116], lacking significant pathogen risk, and potentially generating important data for a broad audience (see Box 1).

**Box 1: Goals for *Pelodera *research**

The utility of studying *Pelodera *is broad, given what is yet to be learned about these species, their ecological roles, and potential for parasitic disease, while also being easy to culture in laboratory settings. Below are a number of potential goals for *Pelodera *research. Use of *Pelodera *specifically is of value due to their parasitic traits, but aspects relating to *Pelodera *have synergies when studied alongside model species or other neglected species.

*Surveillance and Systematics:*

1. Continue surveillance for discovery of novel species of *Pelodera*, particularly in association with insects, during decay of cadavers, and within dermal layers of vertebrates.
2. Generate a genus-wide specimen collection.
  - Improve funding for biobanking and strain maintenance of wild nematodes, including *Pelodera *isolates.
  - Build a specimen database with coupled morphological, molecular, and ecological data.
  - Generate whole mitochondrial genome sequences and rDNA sequences from representative specimens and determine best-use primers / sites for accurate species-specific molecular barcoding.

*Ecology:*

3. Study the ecological benefits of *Pelodera *as part of the succession of decay for (1) animal carcasses, (2) animal faeces, (3) animal nests, and (4) pollution i.e. sewage.
4. Study the ecology and biology of phoresy and necromeny among *Pelodera *vs *Caenorhabditis *and other species capable of necromeny.
5. Determine the relationship between *Pelodera *and beetle hosts as potential commensal or beneficial interactions for decay of animal faeces and carcasses.
  - Determine the impacts of anthelmintics on *Pelodera *in the context of livestock faeces degradation, as part of the wider eco-toxicological impacts of livestock pharmaceuticals.
6. Determine the relationship between *P. strongyloides *and *P. orbitalis *among rodent hosts relating to decay of carcasses, frass, and faeces as well as tissue invasion.

*Developmental genetics and other in vitro analysis*:

7. Improve the available collection and maintenance of *Pelodera *laboratory strains for comparative-analyses and *in vitro *experimentation.
8. Study aspects of *Pelodera *biology that facilitate parasitism including: (1) development of dauer vs infective L3 states, (2) host seeking, (3) tissue invasion (for *larva migrans* capable species), (4) diapause within hosts.
9. Study the dauer pathway of *P. orbitalis *as it compares to *C. elegans *to identify points of divergence between development of dauer, free-living, and parasitic states.
10. Study the environmental requirements and processes facilitating development of L1-L3 stages and perform comparative analyses with obligate parasitic species, particularly Trichostrongylid gastrointestinal nematodes.
11. Determine the capacity for parasitism and variability in infectivity at the strain, species, and genus level in *in vitro *systems including media-based culture and dermal organoids.
12. Transfer genetic techniques from *C. elegans* to *Pelodera *species to functionally validate traits enabling development of infectious L3, host seeking behaviour, and tissue invasion. Determine whether traits are conserved between *Pelodera *and other Clade V parasitic nematodes.
  - Generate RNAi hyper-receptive *rrf-3 *knockout strains of *P. strongyloides*.

*Physiology—Veterinary sciences and medicine*:

13. Anthelmintic discovery of novel compounds that disturb development of infective L3 or host-seeking behaviours using *Pelodera *cultures.
14. Test eco-toxicology impacts of anthelmintics and other veterinary pharmaceuticals (shared with 5a).

*Forensic science*:

15. As in part 5, determine the impact of *Pelodera *on decay of cadavers in relation to host-beetles such as *P. pseudoteres *with *Nicrophorus humator* and the relative disturbance of decay timing when under stress, such as the presence of anthelmintics.

### Pelodera—a potential satellite to triangulate comparative analyses

The idiom ‘variety is the spice of life’ should be applied to non-model nematodes as they can act both as unique points of reference for comparative analyses and as models in their own right. For instance, use of *P. pacificus* to study vulval patterning as a comparator to *C. elegans* resulted in the elevation of this species to a ‘satellite model’ due to being well suited for evolutionary developmental biology [117]. By finding the utility of *P. pacificus* in vulval patterning, interest was generated in studying *Pristionchus* as an intrinsically valuable organism with complex behaviours such as nematode predation and scarab beetle associations [118]. Similarly, research on *Rhabditophanes diutinus*, a free-living Clade IV relative of *Strongyloides*, has highlighted the importance of performing comparative analysis between close relatives, which can otherwise be overlooked [119]. This is because the large evolutionary distance between clades can make broad comparative analyses difficult or unreliable [4, 119–121]. Studying free-living *Pelodera* species can provide similar perspectives to those regarding *P. pacificus* and *R. diutinus* compared to either parasitic *P. strongyloides dermatitica* or further Clade V relatives. The elevation of non-model species demonstrates the potential for *Pelodera* studies to be adopted, while *Pelodera* also can provide unique reference points to data for a wide range of nematological studies, from developmental biology to factors enabling parasitism.

Many aspects of Clade V nematode biology are sadly overlooked, in part because of the diversity of Clade V species. Even for parasitic species, which are well documented, the developmental requirements of free-living stages are poorly documented compared to infectious life stages. Research relating to developmental requirements of free-living stages of parasites is in real need of an update, or even an initial investigation for many species. This need is not just to increase our understanding of parasite biology but also to uncover points of parasite divergence, especially the switch to the infectious L3 state. *Pelodera* species can prove useful as a baseline to understand Clade V parasitism and development in soil. Facultatively infectious species such as Clade IV *Strongyloides ratti* and *S. stercoralis *[122] have been shown to be useful models, especially in the context of comparative analysis with free-living relatives [123, 124]. However, there are still many unknowns in *Strongyloides* biology, including developmental drivers and behaviours dictating parasitic states [56, 125]. *Pelodera* can act as similar models for Clade V parasites and can increase the resolution of comparative analysis by providing nodes among Clade IV *Strongyloides*, Clade V *C. elegans*, and Clade V parasites such as *Haemonchus contortus*.

The conservation of traits that underlie parasitism, especially host invasion, can be triangulated through the inclusion of the parasitic *Pelodera* such as *P. orbitalis*. For *R. diutinus*, some factors that induce dauer larvae were found to be conserved among itself, *C. elegans*, and *Strongyloides* parasites [119], but this knowledge can only be afforded by studying a third species or a collection of species with varying habitats or lifestyles [123, 124]. Being a model organism of extreme importance, comparative analyses already exist between *C. elegans* and many nematode parasites from across the phylum [120], so ‘third’ or n^th^ species may already be available for numerous comparative analyses. However, many parasitic species either lack laboratory models or have extremely complex and costly laboratory models (e.g. intraperitoneal infections of *Brugia malayi* and *Brugia pahangi* in girds as models for human *Wucheraria bancrofti* and *Brugia* lymphatic filariasis infections [126, 127]). *Pelodera* spp. offer a cost-effective format by which to study basic parasitic behaviours, which can be performed at scale and are ripe for transgenesis. Already, non-parasitic species have proved critical for comparative analysis, such as genomic analysis of *P. pacificus*, which revealed a suite of genes involved in detoxification that might facilitate the jump from necromancy to parasitism [128]. In this study *P. pacificus* provided an intermittent node standing between *C. elegans* and distant parasitic relatives such as clade III *B. malayi*. Furthermore, the study revealed the presence of cellulases that had yet to be identified outside of plant-parasitic nematodes and provides more evidence toward the hypothesis of lateral gene transfer between microbiota and nematodes [129, 130]. *P. pacificus* is thus useful for this purpose, but facultatively parasitic *Pelodera* species can go further and add to the wealth of existing information and further detail the evolutionary development of parasitism. The diversity of *Pelodera* habitats can provide reference points to genetic differences underlying free-living, phoretic, necromenic, and parasitic traits. Furthermore, *Pelodera* are also associated with both living and dead plants (Table 1), but little information is available regarding their impacts on plant health, so studying them may aid a better understanding of the roles played at the edge of parasitism in the nutrient cycle of the soil.

To understand animal parasitism, *Pelodera* could offer an improved understanding of parasitic effector production in nematode excretory-secretory (ES) systems. The ES system is a major host-parasite interface with a multitude of functions including production of factors essential for parasitism [131–134]. With ES systems being diverse, their functions in model organisms such as *C. elegans* have been the focus of comparative analysis. The *C. elegans* ES system is composed of four cells (canal, gland, duct, and pore) [135]. The ‘H’-shaped canal cell spans the length of the body that connects posterior to the nerve ring. This channel is linked by a binucleated gland cell, which collectively passes material out of the body through the duct and pore cells [135]. While less described in other species, this system is anatomically conserved in *P. strongyloides* [101]. Being closely related, *Pelodera* species likely contain the same physiology, with subtle differences in parasitic species. Subtle differences could be key to determining factors that enable host invasion for the facultatively parasitic members of *Pelodera* but can also highlight gene families that might play key roles in parasitism in more distant relatives. Again, the reason a cross-comparison with *Pelodera* and *C. elegans* works effectively is their close relationship. In early works of Nematology, Chitwood & Chitwood noted “the diversity of the nemic excretory [-secretory] system makes it a difficult one to interpret” [107]. For instance, even among Clade V, variation is evident, such as in *Ancylostoma caninum* larvae with their large, fused canal and gland cells [136]. Since this structure varies wildly between different nematode clades, identification of parasitic specific behaviours can become obscured when cells and tissues are not analogous and may have multiple differentiated roles. Utilising *Pelodera* to triangulate variance between *C. elegans* and parasitic species will help to build a clearer picture of this tissue, especially among Clade V relatives.

### Technology transfer and functional validation of parasitic behaviours

For Clade V parasites, the pre-adaptation concept (particularly the ‘dauer hypothesis’) is largely accepted as a basis for parasitism. However, there is still a large gap wherein parasite behaviours can be functionally validated. *C. elegans* has often been used to validate parasitic nematode genes [104] and for drug discovery or understanding the mechanism of action [137–139]. Research in *C. elegans* has proved invaluable for understanding fundamental cellular and developmental processes as well as sensory targets of anthelmintics (historically coupled with *Xenopus* oocyte expression systems) [140, 141]. For instance, expressions of *Ancylostoma caninum* or *Cooperia oncophora slo-1* in *C. elegans* mutants are functionally equivalent in locomotion and restore emodepside sensitivity [142]. However, functions in *C. elegans* can differ wildly from other nematodes, even close relatives. For example, the cholinergic agonist target of levamisole (*acr-8*) did functionally predict levamisole sensitivity between *C. elegans* and *H. contortus*, but this was not the case for pyrantel sensitivity [143]. With culture systems for many parasites still lacking, functional validation studies may benefit from inclusion of *Pelodera* or other facultatively parasitic species that are both culturable and receptive to genetic editing techniques. Use of facultative parasites can go one step further than *C. elegans*, given that these species can be cultured effectively in free-living states to generate gene-edited colonies and then assessed in biologically relevant conditions (i.e. infections).

Genetic and genomic techniques for studying gene functions are exceptionally well developed in *C. elegans*. The physiological similarity of *Pelodera* means that there is great promise for adaptation, or even direct transfer of techniques from *C. elegans*. For instance, *Caenorhabditis* and *Pelodera* are gonochoristic, so transgenesis by microinjection into the gonad may be feasible and easier to adapt than in more distant relatives such as *Strongyloides* and [144, 145]. As bacterivores, delivery of compounds via bacterial expression systems, such as for RNAi, may also be a possible avenue to study gene function in *Pelodera*, as was done in *C. elegans* [146, 147] and *Panagrolaimus superbus* [148]. However, for RNAi, this is not always straightforward [149]. Furthermore, assays to determine the impacts of drug targets such as ion channels although pharyngeal pumping could also be utilised in *Pelodera* [150, 151].

Using in vitro analyses in *Pelodera* may make it possible to push and pull parasitic traits in real time or to observe the individual differences between localised parasitic populations (such as for *P. strongyloides*) vs. free-living populations. With genetic tools and culture systems, parasitic traits can be observed in real time and can be aided by high-content imaging and other modern high-throughput technologies, e.g. for studying free-living vs. parasitic sensory biology or anthelmintic resistance [152, 153].

Already, the availability of the *P. strongyloides* transcriptome has shown that *P. strongyloides* expresses key components of the RNAi pathway alongside documented repressors of RNAi such as Dicer, Argonaute, RNA-dependent RNA polymerase, dsRNA transporters (e.g. SID-like proteins), and repressors such as *rrf-3* [48]. It is possible to utilise this information to probe the efficacy of RNAi in *Pelodera* and rapidly generate hyper-responsive *Pelodera* through knockdown or knockouts of RNAi inhibiting genes such as *rrf-3* [146, 154].

Another requirement for in-depth laboratory analyses is the ability to culture and maintain worms in vitro or in highly tuneable environments where genotype can be aligned to phenotype. *Pelodera*, being easy to culture in the absence of hosts, can provide useful models in this instance [57, 115, 116]. Advances in single worm sequencing also mean that the time may be ripe to trace divergent states [155], and we could use this technology to profile differences at the species level among facultative parasites such as *P. orbitalis*, which have the benefit of being culturable.

### Anthelmintic drug discovery and ecotoxicology

In addition to high-capacity techniques such as gene-editing and sequencing, simple assays can be performed which have great value in identifying novel compounds on anthelmintics. Use of *Pelodera* for anthelmintic discovery has two main points of consideration. Point 1 is that novel anthelmintics should be tailored to disrupt parasitism specifically and are ideally species-specific and non-toxic. Therefore, use of *Pelodera* alongside *C. elegans* and other obligate parasitic L3 species could help delineate the ecotoxicology of available and novel anthelmintics. This is especially important in the context of monitoring the impacts of anthelmintics used in the livestock industry, since *Pelodera* thrive as faecal decomposers and are closely associated with dung beetles. Studying the impact of anthelmintic use in real-world conditions is an area of research that needs improved monitoring with relevant species. Evidence of dung-beetle susceptibility to ivermectin has long been apparent, but what happens to the fitness of dung beetles and other insects in cases where free-living saprotrophic nematode species are killed?

Point 2 is the potential to identify novel anthelmintics using *Pelodera* as a stand-in for obligate parasites, since it is easy to culture while retaining a parasitic state. Therefore, *Pelodera* can be applied in studies that can disturb the formation of infectious L3 or studies that impair ‘infectious’ behaviours such as motility and host-seeking for parasite-specific nematicides and anthelmintics. In this case, novel anthelmintics identified will inadvertently be lethal to *Pelodera*, which counteracts point 1. However, there is a dearth of available new anthelmintics, with available compounds losing efficacy in the face of anthelmintic resistance among nematode parasites of livestock [158, 159]. Furthermore, some compounds, such as ivermectin, have a broad spectrum of activity, so the development of novel anthelmintics has a relatively low bar to cross to make improvements. Again, compounds could be tested that are ecologically safe and fail to kill non-infectious *Pelodera* or only target obligate parasitic nematodes but fail to kill dung beetles and their associated saprophytic nematodes. Any compounds identified that are extremely potent could still be useful as part of the collective anthelmintic arsenal, needed for cases where multiple anthelmintic failure is an issue.

## *Pelodera* as models for parasitic origins

When studying the origins of parasitism, questions arise about what adaptations make particular nematodes more amenable to parasitism than others. If leaf litter, humus, and surface substrates, such as windfall apples, are a perfect environment for terrestrial species such as *C. elegans* [160, 161], then why is *C. elegans* not a parasite while relatives such as *P. orbitalis* and *P. strongyloides* are? Closer ‘parasitic’ relatives to *C. elegans* exist, such as *C. bovis* (in inflamed ears of zebu cattle [100]) and *Caenorhabditis avicola* (observed in the intestine of a passerine bird [162]). These potentially tenuous populations of *Caenorhabditis* parasites are possibly niche specialists, possibly the result of recent plasticity among free-living generalists. More work is needed to clarify the true range of habitats in which cosmopolitan species can colonise. We know that *P. cystilarva* is specific to endophoresy within ant heads, a host association that cannot be replicated by *P. strongyloides* [72]. More examples and experimentation are needed to determine the capacity for parasitic species to cause infections like follicular larva migrans. In addition we need to ask what variations in virulence exist among populations of parasitic species.

### Origins and discovery of parasitic traits

With nematodes identified in almost every habitat on Earth, it is perhaps unsurprising that parasitism as a lifestyle has arisen numerous times among nematodes for vertebrates, invertebrates, and plants, with vertebrate parasites present across Clade I (Dorylaimia), Clade III (Spiruina), Clade IV (Tylenchina), and Clade V (Rhabditina) [120, 163]. With such a spread of vertebrate parasitism across the phylum, parasitism is considered a homoplasious trait, emerging independently from terrestrial origins at least five times [163–165]. Similarly, at least 10 origins of invertebrate parasitism are thought to exist [163]. Terrestrial habitats are considered the main context in which parasitic behaviours emerge [32, 163, 164]. Points of parasite origin have been determined for a number of nematode clades, but there is much to be learned relating to the processes enabling the emergence of parasitism, especially among Clade V species. This is partly due to the vast number of Clade V parasitic species and their diversity (Rhabditida contains the largest collection of parasitic species) but also due to variance in associations between Clade V nematodes and other metazoans, particularly invertebrates and mammals [28, 166].

For nematodes to make the transition from free-living to parasitic states requires overcoming a number of barriers to infection, such as survival at higher temperatures, tolerance of acidic or oxygen-depleted environments, immune evasion and resistance to digestive enzymes, as well as abilities of offspring to migrate out of hosts and for the next generation to detect new hosts [32]. The presence of distinct parasitic lineages suggests that certain nematodes may be able to overcome these barriers [163]. Since many genera contain both parasitic and free-living species, other factors such as pre-existing physiological traits facilitating parasitism alongside habitat sharing with hosts are also required to provide opportunities for parasitism to arise [32]. However, survival in and amongst the soil is varied and requires diversification of traits to survive compared to host niche environments. The soil is also a pivotal part of the life cycle for many Clade V parasitic nematodes including hookworms and Trichostrongylid gastrointestinal nematodes [167, 168]. Interactions with the soil and climatic conditions outside of the host largely dictate parasite developmental progression and host-seeking behaviours that dictate successful colonisation of new hosts.

It is probable that many obligate nematode parasites were once free-living faecal decomposers of their mammalian hosts. If that is the case, then *P. strongyloides* may be in an early transitional stage toward parasitism. For instance, a number of parasites including hookworms and Trichostrongylid gastrointestinal nematodes of livestock are dependent on nutrients from faeces or soil to mature from L1 to infectious L3, but research in this area is in real need of renewal [167–170]. The degree of consumption between egg and infective L3 varies, and subsistence is not necessary for some, such as the cattle lungworm *Dictyocaulus viviparus* [171]. Nevertheless, parasite development in faeces or soil is more than a historical remnant of ‘free-living’ lifestyles, since for these species, development and persistence outside of the host are critical to infecting new hosts, and as such are part of the spatial dynamics of infection. *Pelodera* species may offer a simple means to improve our understanding of developmental cues in saprophytic environments as easy-to-culture organisms [57, 116], while being closely associated with hosts and having inducible parasitic states [30, 39, 172].

For some *Pelodera* species, endophoresy and necromeny are sufficient to be parasitic, causing dermatitis despite not benefiting from host invasion directly [172]. For more established parasites, the degree of migration, development, host resource deprivation, and virulence varies. What unifies the move toward parasitising hosts is environmental stress. Parasitism might be the result of long-term environmental stress, wherein once free-living species find better resources in a host or are simply the result of successful migration tactics to move toward more fertile soil. The single-generation environmental stressors which induce parasitic phenotypes are known for entomopathogenic nematodes [28]. In inducible *P. orbitalis*, sensory behaviour is largely unknown. For other facultative parasitic species, most notably Clade IV *Strongyloides*, the environmental stress is temperature, food quality, and quantity; however, more study is needed to unravel cues dictating development of parasitic states [173].

Overall, there is a likelihood that *Pelodera* are sitting at the interchange between free-living and parasitic lifestyles, and it is probable that studying *Pelodera* behaviours will help in the elucidation of genetic switches that can guide parasitic vs. free-living states of being.

### Gene expansion, a result of saprophytic lifestyles or of host colonisation?

Free-living, saprophytic lifestylesprobable require a high level of adaptations that can prepare for parasitic behaviours. Chemoreceptors stand out as prime examples, being most abundant and diverse amongst free-living species while gradually declining amongst parasitic species relative to their degree of environmental exposure [174]. Species with the lowest total chemoreceptors are those which spend little to no part of their life cycle interacting with the environment outside of their hosts (e.g. Clade I *Trichinella* and *Trichuris* and Clade III vector-transmitted filaria). Meanwhile, parasites which spend a portion of their lives in terrestrial environments (usually during L1–L2 development) have retained a large portion of their chemoreceptors, such as Clade V gastrointestinal parasites, hookworms, and Clade IV *Strongyloides*. For all parasitic nematodes, we can imagine that their ancestors shared similar habitats with free-living counterparts. It is likely that the more ancient vertebrate parasites (such as Oxiurids [175, 176]) have diminished their repertoire of chemoreceptors over time while free-living specialists have expanded.

Gene expansion is a well-documented precursor to spillover events or enhanced colonisation of hosts amongst a diverse array of pathogens [177]. Famous and extreme examples exist, such as *Phytophthora infestans* (potato blight oomycete), which underwent rapid and massive genome expansion that facilitated diversification of secreted effector proteins [178]. Nematodes can take advantage of the faster rates of evolution among microbes through horizontal gene transfer of factors that facilitate plant parasitism [129, 130, 132, 163, 179] or environmental stress tolerance [180]. In nematodes, there is a significantly slower evolutionary time, but expansion of gene families associated with parasitism still occurs. For plant-parasitic nematodes, SPRY and other gene families have undergone massive expansion as part of a plant-nematode arms race [181, 182]. Examples also exist for vertebrate parasites, such as steroid kinase family expansions among clade IV *Strongyloides* and *Bursaphelenchus*, which may have either arisen because of the pressures of post-pathogen interactions or facilitated the switch to parasitism [120]. It can be difficult to determine the order of events, whether expansion came first and aided colonisation or whether the selection pressure of colonisation prompted gene expansion. A number of parasite-specific gene family expansions exist among exclusively parasitic lineages of vertebrate parasites, such as proteases and protease inhibitors [120]. These indicate that pre-existing traits may not always be sufficient for parasitism, but that expansion of effectors is still part of the evolutionary journey toward obligate parasitism. Overall, each of these above examples point to wildly different aspects of parasite biology. In reality, there is an ever-shifting diversity of traits that in combination with opportunities and random chance facilitates transition to parasitic lifestyles, followed by selection pressure and expansion of specific families at the expense of other traits as part of host-niche specialisation. One thing is for certain, however, which is the need to study more free-living and facultative parasites to improve our overall understanding and perspective on parasite behaviours. The International Helminth Genomes Consortium authors note that resolving parasitic functions requires greater genetic resources from free-living species [120].

### Lane switching, can parasites revert to free-living lifestyles?

Further study is needed to determine the evolutionary time of parasitic origins for many vertebrate parasites, and similarly more work is needed to determine whether parasitism decreases overall fitness for varied environments the more they become specialised to host niches. We do know that parasitism is not strictly a one-way street, with multiple rounds of parasitic behaviours being gained and lost for plant parasitic nematodes and their invertebrate associates [183]. The discovery of a free-living clade III nematode suggests that parasitic behaviours can be lost in tissue-embedded vertebrate parasites as well [184]. However, Nadler et al. show through re-analysis that these unusual filaria are probably *Tetrameres* or similar avian parasites and may simply have been deposited by infected birds rather than being truly free-living [165]. This leaves us with little evidence of vertebrate parasites returning to free-living lifestyles, although this is an area of considerable research interest due its potential for control applications. For instance, there is interest in altering hypobiotic states to induce exsheathment of infectious stage L3 larvae of strongyles to induce development outside of the host, preventing infections. It appears that abiotic factors (pH, temperature, CO_2_) dictating exsheathment are highly species and context specific for a wide range of ruminant gastrointestinal nematode species [185]. Biological factors may be more conserved, however, with much of the *C. elegans* dauer pathway conserved across vertebrate parasites, although the presence of a dauer or quiescence state varies wildly between species, and the pathways directing this development are far from resolved [34].

Understanding biological processes at the interchange between free-living and parasitic states is necessary to deconvolute data from obligate parasites. For instance, Clade V Trichostrongylid gastrointestinal nematodes of livestock are dependent on nutrients from faeces or soil to mature from L1 to infectious L3, but research in this area is in real need of renewal [169, 170]. To what extent is the free-living stage of a parasite a free-living species in of itself? Is it possible to induce a state where development proceeds outside of the host? Many of these species are relatively straightforward to culture through to fecund adults in simple in vitro conditions (such as *Ostertagia ostertagi* in media containing chicken embryo extract) [186], so this may not be impossible if large scale gene editing technologies can be mastered in obligate parasites. In the meantime, use of *Pelodera* or facultative parasites could fill this niche. *Pelodera*, which exists on the boundary between free-living and parasitic, also lies in contrast to the specific host environments needed to replicate life cycles through model host systems or host-tissue organoids [111]. Complex, host-mimicking environments may be on the horizon, but in many cases we lack basic understanding of the requirements necessary to induce the progression of development.

Again, identifying cues from free-living species may provide context to improve the functionality of in vitro systems, especially during development [40]. Similarly, with improved understanding, it may be possible to hijack some obligate parasites and induce continued development ex vivo in the environment. The reward of doing so will be to develop potential novel therapeutics that inhibit development or stimulate overdevelopment of parasitic larvae in the environment.

## Conclusions

*Pelodera* is a widespread and populous genus of saprotrophic nematodes, frequently identified as a dominant taxon among soils, decomposing animal carcasses, nesting sites, and faeces. *Pelodera* likely plays a major but underappreciated role in nutrient cycling among animals, plants, and the soil. Part of the success of *Pelodera* among soils and decomposing organic matter comes from their use of invertebrate and vertebrate hosts for translocation to new areas, including some extremely isolated areas such as littoral regions of the Arctic and Antarctic islands. Host associations among different species of the genus are varied and range from ranging from free-living (i.e. no known hosts) to parasitic. Most species exhibit dauer larvae or infectious L3 that seek and attach to invertebrate and mammalian hosts, especially dung beetles and rodents. Some *Pelodera* species are known to have inducible parasitic L3 states and thus have developmental plasticity to engage in parasitism or phoresy when conditions require movement to new areas. While infections of rodents are apparently well tolerated, infections of other mammals can cause distressing disease, most notably *P. strongyloides*, which can cause follicular cutaneous larva migrans in a wide range of mammals. There is therefore great value in further study of this genus, not only to delineate beneficial impacts on soil health but also to determine their impacts on insect ecology and mammalian ecology as part of necromenic commensalists and parasites of the skin. Investment and interest in studying this group of nematodes will also build on our understanding of parasitism in nematodes and the behaviours and conditions in which parasitism arises.

## Data Availability

Data supporting the main conclusions of this study are included in the manuscript.
